# Obituary—W. R. Marsh, M. D.

**Published:** 1873-12

**Authors:** 


### *Obituary.*

WELLS RANSOM MARSH, A.M., M.D., died October 15th, 1873, at Crystal Springs, Yates county, New York, of *Pneumonia Gangrænosa.*

Dr. Marsh was born in Townshend, Vermont, A. D. 1826. The family migrated to Kalamazoo, Michigan, in 1833, being among the pioneers of that section of the State. Developing a strong taste for study, the subject of our sketch entered the University at Ann Arbor, and graduated with honor in 1848. He subsequently received the degree of A.M. from the same institution. He attended medical lectures at the Indiana Medical College, and then located at Laporte, having the diploma of M.D. granted him at the last session of the college in that place.

After practicing his profession for several years in Kalamazoo, he was solicited, in connection with his brother, Charles P. Marsh, M.D., to take professional charge of the large colony of Hollanders who, under the care of the Rev. A. C. Van Raalte, located at Holland and its vicinity, on the eastern shore of Lake Michigan. Probably no severer, more arduous or harassing toil ever fell to the lot of two physicians, than this position imposed on the brothers Marsh. The sole medical, surgical and obstetrical charge of some six thousand persons, suddenly collected in what was then an unbroken forest, devolved upon them. With what untiring assiduity, sleepless care and conscientious zeal the brothers discharged their duty there, none but those personally cognizant of the facts can appreciate. The feeble colony has become a prosperous and wealthy community, as it always was an industrious and intelligent one, and no hearts and memories will be filled with sincerer grateful recollections, and more lasting reminiscences of respect and love, than theirs at the mention of the names of the Drs. Marsh.

In 1856, the subject of this notice received the appointment of Professor of Chemistry and Materia Medica in the Medical College at Keokuk, Iowa, a chair which he filled until the beginning of the late war, when he entered the service as Surgeon of the 2d Iowa Volunteers, under the first three months call, being thus the second in point of seniority of the volunteer surgeons of that State, and at the time of being mustered out in 1865, he ranked as oldest in commission of the volunteer surgeons of the West. During the entire war, except when temporarily disabled by sickness, he was in the most active service, being present at nearly all the principal skirmishes, great battles, and severest marches and expeditions from Belmont and Fort Donaldson to Atlanta and Savannah, then through the Carolinas, to Richmond, Washington, and "home again."

Enfeebled in health and broken in constitution, by the continuous hardships to which his campaign life had subjected him, "when the cruel war was over" he settled for practice in Chicago, where he rapidly gained friends and the respect of his professional acquaintances. But the worn and disabled physical part refused to rally, and some two years since he went East, if possible, to recuperate. Whilst there he accepted the sanitary charge of the Crystal Springs establishment, and not only won the hearts of his immediate patients, but was fast gaining the confidence of the physicians in the country about, by whom he was frequently consulted. Here he was suddenly struck down by the disease which his shattered constitution, although for a long period it had been most cautiously cared for, could not resist.

In person Dr. Marsh was of commanding figure, about six feet and an inch in height, well and symmetrically proportioned, straight as an arrow, black hair and keen black eyes. Fluent in conversation, quick to discern and ready in response, he was always the life of the circle where he was present. As a practitioner he was far above the average—an excellent diagnostician, usually a very cautious, but on occasion a bold therapeutist or operator. As a lecturer he was characterized by his thorough command of the subject, his obviously earnest desire that the student should understand his teachings, and by his cultivated, scholarly style of discourse.

Dr. Marsh acted for some time as editor of the Keokuk (Iowa) *Medical Journal*, and has contributed valuable papers to other periodicals. A report from his pen on the cholera in Chicago, in 1866, was read at the annual meeting of the Illinois State Medical Society at Springfield, in 1867, and will be remembered as an important contribution to the literature of the subject.

He leaves, besides a large circle of relatives and friends, a wife and four children to mourn his loss.

